# Helminth community structure of *Didelphis marsupialis* (Didelphimorphia, Didelphidae) in a transition area between the Brazilian Amazon and the Cerrado

**DOI:** 10.1590/S1984-29612022031

**Published:** 2022-06-06

**Authors:** Leodil da Costa Freitas, Arnaldo Maldonado, Ravena Fernanda Braga de Mendonça, Dirceu Guilherme de Souza Ramos, Rogério Vieira Rossi, Richard de Campos Pacheco, Rosana Gentile

**Affiliations:** 1 Laboratório de Parasitologia Veterinária e Doenças Parasitárias dos Animais Domésticos e Silvestres, Faculdade de Medicina Veterinária – FAVET, Universidade Federal de Mato Grosso – UFMT, Cuiabá, MT, Brasil; 2 Laboratório de Biologia e Parasitologia de Mamíferos Silvestres Reservatórios, Instituto Oswaldo Cruz – FIOCRUZ, Rio de Janeiro, RJ, Brasil; 3 Laboratório de Mastozoologia, Instituto de Biociências – IB, Universidade Federal de Mato Grosso – UFMT, Cuiabá, MT, Brasil; 4 Laboratório de Patologia e Parasitologia Veterinária, Unidade Acadêmica de Ciências Veterinárias, Universidade Federal de Jataí - UFJ, Jataí, GO, Brasil; 5 Programa de Pós-graduação em Ecologia e Conservação da Biodiversidade – PPG/ECB, Instituto de Biociências – IB, Universidade Federal de Mato Grosso – UFMT, Cuiabá, MT, Brasil

**Keywords:** Amazonia, Brazil, Nematoda, Acanthocephala, parasite ecology, parasitism, Amazônia, Brasil, Nematoda, Acanthocephala, ecologia de parasitos, parasitismo

## Abstract

Although the common opossum, *Didelphis marsupialis* (Didelphimorphia: Didelphidae) is a species widely distributed in South America, knowledge about their helminth parasites and helminth community structure is scarce. The aims of this study were to describe the species composition and analyze the structure of the helminth community of the common opossum in an area of the Amazonian Arc in northern Mato Grosso. The helminths were recovered, counted, and identified in 32 individuals. Overall, 10,198 specimens were categorized into 9 helminths taxa (seven nematodes, one cestode, and one acanthocephalan). The most abundant species were *Aspidodera raillieti*, *Viannaia hamata*, and *Travassostrongylus orloffi*. No statistically significant differences in helminth abundance and prevalence were observed between host sexes. However, young hosts had higher abundance and prevalence of *Didelphonema longispiculata*, whereas *Oligacanthorhynchus microcephalus* had higher abundance and prevalence in adult hosts. This was the first study to analyze the helminth fauna and helminth community structure of *D. marsupialis* in the Amazonian Arc. This is the first report of the presence of *A. raillieti*, *D. longispiculata*, *T. orloffi*, *T. minuta*, *V. hamata*, and *O. microcephalus* in the state of Mato Grosso, Brazil.

## Resumo

Embora o gambá comum, *Didelphis marsupialis* (Didelphimorphia: Didelphidae) seja uma espécie amplamente distribuída na América do Sul, o conhecimento sobre seus helmintos parasitos e a estrutura da comunidade de helmintos são escassos. Os objetivos deste estudo foram descrever a composição de espécies e analisar a estrutura da comunidade helmíntica do gambá comum em uma área do Arco Amazônico, no norte de Mato Grosso. Os helmintos foram recuperados, contados e identificados em 32 indivíduos. Ao todo, 10.198 espécimes foram categorizados em 9 táxons de helmintos (sete nematoides, um cestoide e um acantocéfalo). As espécies mais abundantes foram *Aspidodera raillieti*, *Viannaia hamata* e *Travassostrongylus orloffi*. Não foram observadas diferenças estatisticamente significativas na abundância e prevalência de helmintos entre os sexos dos hospedeiros. No entanto, hospedeiros jovens apresentaram maior abundância e prevalência de *Didelphonema longispiculata*, enquanto *Oligacanthorhynchus microcephalus* apresentou maior abundância e prevalência em hospedeiros adultos. Este foi o primeiro estudo a analisar a helmintofauna e a estrutura da comunidade helmíntica de *D. marsupialis* no Arco Amazônico. Este é o primeiro relato da presença de A. *raillieti*, *D. longispiculata*, *T. orloffi*, *T. minuta*, *V. hamata* e *O. microcephalus* no estado de Mato Grosso, Brasil.

## Introduction

Helminths usually show a variety of transmission patterns that are determined by intrinsic factors such as their biological cycle characteristics and environmental requirements (Mas-Coma et al., 2008). The relative importance of these biotic and abiotic factors is not fully understood in most host–parasite interactions. Seasonal variations in temperature, humidity, and host characteristics, such as food habits, habitat preference, age, weight, sex, and body size, can regulate the dynamics (biological cycle) of parasitism within the host, and are often examined in ecological studies of many parasites (Ferrari, 2005; Krasnov et al., 2005; Simões et al., 2014). These factors can determine host–parasite contact and thus influence the dynamics of the parasite population, the spatial distribution of the parasite, and the risk of infection in a given host (Bush et al., 2001; Altizer et al., 2006).

Among marsupials, the common opossums have a special interest because they are reservoirs of several zoonosis, such as trypanosomiasis, leishmaniosis, and helminthiasis (Lima et al., 2012; Jansen et al., 2015; Costa-Neto et al., 2016; Bezerra-Santos et al., 2021). Among host mammals, male specimens tend to have a greater abundance, prevalence, and species richness of helminths than females (Poulin, 1996; Schalk & Forbes, 1997; Soliman et al., 2001; Rossin & Malizia, 2002). These sex-related trends in host mammals have been correlated with behavior and habits, as well as with hormonal androgen levels, differences in body mass and size, and physiological stress levels (Arneberg et al., 1998; Moore & Wilson, 2002; Morand et al., 2004; Krasnov et al., 2011).

Likewise, older hosts may have higher parasite loads because of opportunities for greater exposure and contact with parasites throughout their lives (Anderson & Gordon, 1982; Anderson & May, 1991; Hudson et al., 2002; Cooper et al., 2012).

In Brazil, 62 marsupial species have been identified (Faria et al., 2019), but their helminth fauna is still poorly studied. The majority of helminth studies on Brazilian mammals consist only of reports of helminth species records (Gomes et al., 2003; Thatcher, 2006; Cardia et al., 2016) and taxonomic descriptions (Torres et al., 2007; Adnet et al., 2009; Araújo, 2011; Chagas-Moutinho et al., 2007; Chero et al., 2017). However, few studies have been conducted on the helminth community structure of neotropical marsupials (Silva & Costa, 1999; Antunes, 2005; Jiménez et al., 2011; Zabott et al., 2017; Costa-Neto et al., 2019; Ramírez-Cañas et al., 2019; Cirino et al., 2020; Cirino et al., 2022).

Previous studies on the helminths of the common opossum, *Didelphis marsupialis* Linnaeus, 1758 were mainly taxonomic descriptions and occurrence records (Vicente et al., 1997; Rodriguez-Ortíz et al., 2004; Acosta-Virgen et al., 2015; Thatcher, 2006; Chagas-Moutinho et al., 2014, Chero et al., 2017), with a single study examining the helminth communities of didelphid marsupials, including common opossum (Jiménez et al., 2011).

The aims of this study were to describe the species composition and to analyze the structure of the helminth community of *D. marsupialis* at the infracommunity level (i.e., within an individual host) and at the component community level (i.e., at the level of a set of infracommunities of a given host population) in an area of the Amazonian Arc in northern Mato Grosso. Furthermore, we also tested whether host sex and age were determining factors for helminth parasitism in this marsupial.

## Materials and Methods

### Study area

**Figure 1:**
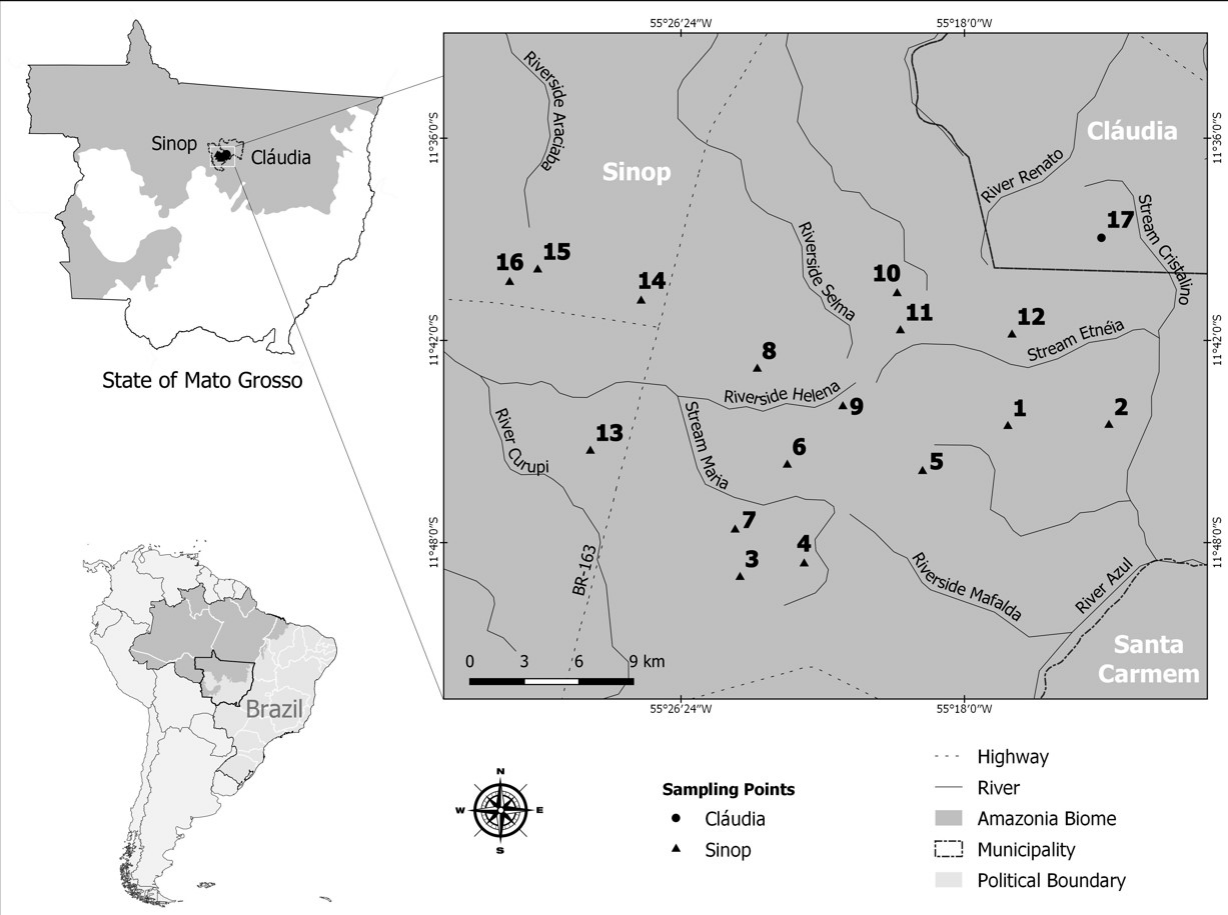
Map showing the sampling transects where *Didelphis marsupialis* captures were performed in the municipalities of Sinop and Cláudia within Amazonian Arc in northern Mato Grosso State, Brazil.

This study was carried out on 17 sample transects installed in forest fragments in the municipalities of Sinop (11° 491.71″ S, 55° 2439.05″ W) and Claudia (11° 3055″ S, 54° 5329″ W) (Figure 1). These fragments, with sizes ranging from 81.7 to 19,838 ha, were delimited by an agricultural area located in southern Amazonia in a transition zone with the Cerrado biome, in the state of Mato Grosso, Brazil (Figure 1). The region's climate is tropical hot and humid (Aw) with monsoon-like rains, in transition with the equatorial super humid climate (Am) of the Amazon (Alvares et al., 2013). The average annual precipitation is 2,000 mm, with two well-defined seasons: a rainy season (from October to April) and a dry season (from May to September), with average annual temperatures ranging between 24 °C and 27 °C (Priante-Filho et al., 2004).

### Sample collection method

This study was approved by the Ethics Committee on the Use of Animals (CEUA) of the Federal University of Mato Grosso (UFMT) under protocol nº 23108.076870/2015-41 approved on April 16, 2015. The study was also approved for a permanent license, for the collection of zoological material to study small non-flying mammals, by the Chico Mendes Institute for Biodiversity Conservation (ICMBio, license nº 8863-1).

Marsupials had been captured primarily with the aim of studying the ectoparasite-host interaction network (Mendonça et al., 2020). Briefly, a 300 m long transect was established at each sampling point, excluding 100 m from the edge of the study area, where 30 capture stations were installed 10 m equidistant from each other. At each prospected point, 60 traps for conventional capture of small non-flying mammals were installed, 30 of the Sherman type (9.5 × 8 × 25 cm) and 30 of the cage type (16.5 × 16.5 × 35 cm), arranged alternately on the ground and understory (with a height of at least 1.5 m). A mixture of peanut butter, cornmeal, sardine, and vanilla flavoring added to a piece of banana was used as bait. The traps were set for eight consecutive nights, checked daily, and baited again whenever there was a need to replace the bait or every three days. The sampling effort of each transect was 480 trap-nights, totaling all 17 transects, resulting in a total of 16,320 trap-nights, considering two expeditions, one during the rainy season (November 2016) and the other during the dry season (July 2017).

The captured marsupials were anesthetized for screening and identification based on Gardner (2007). The animals were weighed using 500 g and 1000 g Pesola® brand scales (the weights of individual animals above 1000 g were estimated), measured with a 100 cm measuring tape. After this step, euthanasia was performed by increasing the anesthetic dose, according to the standards established by the Federal Council of Veterinary Medicine. All individuals were submitted to taxidermy, and voucher specimens were deposited in the Zoological Collection of the Federal University of Mato Grosso, Cuiabá, Mato Grosso State, Brazil (Appendix 1)

### Helminth collection, fixation, and identification

The gastrointestinal material of each animal was removed immediately after the animals were euthanized and placed in plastic pots containing a 70% ethanol-based preservative liquid. Subsequently, organs were separated in Petri dishes and dissected using a stereoscopic microscope for helminth recovery. The specimens were stored in glass tubes and properly labeled with host data and the sampling points in which they were trapped.

The collected helminth specimens were washed in physiological solution and preserved in 70% ethanol to remove tissue debris. Some of the helminth samples were fixed in AFA solution (93 parts 70% ethanol, 5 parts 0.4% formol and 2 parts 100% acetic acid) and heated to 65° C, while other samples were kept in 70% ethanol for molecular analysis.

For light microscopy, helminth specimens from ten male and ten female host animals were clarified in lactophenol/phenol at 50%, placed on temporary slides, and examined using a standard 20 Zeiss light microscope. Both cestodes and acanthocephalans were stained with Carmine of Langeron, following standard protocols (Amato et al., 1991).

Specific morphological aspects were used to identify the specimens, based on studies by Travassos (1939), Yamaguti (1961), Vicente et al. (1997), and Anderson et al. (2009), as well as on articles describing related species. All helminths were identified at the Laboratory of Biology and Parasitology of Wild Mammals Reservoirs of the Oswaldo Cruz Institute (IOC), located at the Oswaldo Cruz Foundation (Fiocruz), Rio de Janeiro (RJ), Brazil. The specimens were deposited in the scientific collection of helminths at the Laboratory of Biology and Parasitology of Wild Mammals Reservoirs-IOC/Fiocruz-RJ (Appendix 1).

### Data analysis

The community structure of this parasite was studied at the infracommunity and component community levels (Bush et al., 1997). The total helminth species richness was defined as the number of species found. The mean species richness was defined as the number of helminth species in each infracommunity divided by the number of hosts analyzed. The estimated species richness was calculated using the Jackknife 1 method (Magurran, 2011).

Mean abundance, mean intensity, and prevalence were calculated according to Bush et al. (1997). The mean abundance was calculated as the total number of individuals of a given helminth species divided by the number of analyzed hosts. The mean intensity was defined as the total number of individuals of a helminth species divided by the number of hosts infected with this species. The prevalence of each helminth species was calculated as the proportion of infected hosts for a given helminth species in relation to the total number of analyzed hosts. The influence of host sex and age on abundance was tested using the Mann–Whitney test. The influence of host sex and age on prevalence was tested using the chi-square test.

The influence of host body size on the total abundance and species richness for each infracommunity was investigated using linear regression, separately for each host sex. The significance of the regression coefficient (beta) was evaluated using t-tests.

The importance indices of each helminth species were calculated according to Thul et al. (1985). Each helminth species was classified in the community as dominant (I ≥ 1.0), co-dominant (0.01 ≤ I < 1.0), or subordinate (0 <I < 0.01).

All analyses were performed using the Past software version 3.21 (Hammer et al., 2001). The data were tested for normal distribution using the Shapiro–Wilk test. A significance level of 5% was considered for all analyses.

## Results

In total, 32 common opossums were captured and analyzed, with eight males and 24 females, and 19 young and 13 adults. Of these, 31 marsupials were parasitized with at least one species of helminth. Overall, 10,198 helminths were recovered and categorized into 9 helminths taxa (seven nematodes, one cestode, and one acanthocephalan), identified as follows (with the number of specimens): *Aspidodera raillieti* Travassos, 1913 (Ascaridida, Aspidoderidae) (6,756); *Cruzia tentaculata* Rudolphi, 1819 (Ascaridida, Kathlaniidae) (4); *Didelphonema longispiculata* Hill, 1939 (Spirurida, Spirocercidae) (110); *Travassostrongylus orloffi* Travassos, 1935 (Rhabditida,Viannaiidae) (624); *Trichuris minuta* Rudolphi, 1819 (Trichocephalida, Trichuridae) (22); *Turgida turgida* Rudolphi, 1819 (Spirurida, Physalopteridae) (61); *Viannaia hamata* Travassos, 1914 (Rhabditida,Viannaiidae) (2,190); *Oligacanthorhynchus microcephalus* Rudolphi, 1819 (Archiacanthocephala, Oligacanthorhynchidae) (75); and a species of Cestoda, which was not identified at specific level due to the absence of diagnostic taxonomic characters (356).

The most abundant species was *A. raillieti*, followed by *V. hamata* and *T. orloffi*, while *A. raillieti* was also the most prevalent (Table 1). The helminth richness ranged from 0 to 9, with a mean of 4.3 species per host, and with no difference between the total richness observed and that expected (Jackniffe 1 = 9).

**Table 1:** Mean abundance, mean intensity of the helminths with standard deviation, and helminth prevalence (%) with 95% confidence limits in relation to host sex and age found in *Didelphis marsupialis* (N = 32; N_male_ = 8; N_female_ = 24; N_young_ = 19; N_adult_ = 13) from Mato Grosso State, Brazil.

**Parameters**	***Aspidodera raillieti***	***Cruzia tentaculata***	***Didelphonema longispiculata***	***Travassostrongylus orloffi***	***Trichuris minuta***	***Turgida turgida***	***Viannaia hamata***	**Cestoda**	***Oligacanthorhynchus microcephalus***
**Abundance**	211.13±217.55	0.13±0.42	3.44±6.55	19.50±34.14	0.69±1.28	1.91±2.36	68.44±131.64	11.13±62.93	2.34±3.29
Male	150.38±153.10	0.00±0.00	5.25±11.74	12.75±15.84	1.13±1.81	1.50±2.83	38.75±50.09	0.00±0.00	1.50±2.33
Female	231.38±234.40	0.17±0.48	2.83±3.78	21.75±38.39	0.54±1.06	2.04±2.24	78.33±148.95	14.83±72.67	2.63±3.78
Juvenile	144.79±120.28	0.11±0.46	5.11±7.93	25.47±42.43	0.95±1.51	1.58±2.41	94.89±163.67	0.00±0.00*	1.57±3.32
Adult	308.08±288.81	0.15±0.38	1.00±2.38	10.77±13.10	0.31±0.75	2.38±2.29	29.77±43.09	27.38±98.74	2.85±3.21
**Intensity**	217.94±217.62	1.33±0.58	7.86±8.06	27.13±37.76	2.20±1.40	3.21±2.27	95.22±147.49	356.00±*	4.69±3.26
Male	150.38±153.10	0.00±0.00	10.50±15.75	20.40±15.61	3.00±1.73	4.00±3.61	62.00±50.88	0.00±0.00	4.00±2.00
Female	241.44±234.31	1.33±0.58	6.80±2.57	29.00±42.08	1.86±1.21	3.06±2.08	104.44±164.70	356.00±*	4.85±3.53
Juvenile	144.79±120.28	2.00±*	8.82±8.78	37.23±47.18	2.25±1.58	3.33±2.55	120.20±176.59	0.00±0.00	5.29±3.54
Adult	333.75±285.74	1.00±0.00	4.33±3.51	14.00±13.36	2.00±0.00	3.10±2.13	48.36±46.41	356.00±*	4.22±3.15
**Prevalence^¥^**	96.90	9.40	43.80	71.90	31.30	59.40	71.90	9.40	50.00
	(83.80-99.90)	(2.00-25.00)	(26.30-62.30)	(53.30-86.30)	(16.10-50.00)	(40.60-76.30)	(53.30-86.30)	(2.00-25.00)	(31.90-68.10)
Male	100.00	0.00	50.00	62.50	37.50	37.50	62.50	12.50	37.50
	(63.06-100.00)	(0.00-36.10)	(15.70-84.30)	(25.00-91.50)	(8.50-75.50)	(8.50-75.50)	(24.50-91.50)	(0.30-52.70)	(8.50-75.50)
Female	95.80	12.50	41.70	75.00	29.20	66.70	75.00	8.30	54.20
	(78.90-99.90)	(2.70-32.40)	(22.10-63.40)	(53.30-90.20)	(12.60-51.10)	(44.70-84.40)	(53.30-90.20)	(1.00-27.00)	(32.80-74.50)
Juvenile	100.00	5.30	57.90	68.40	42.10	47.40	79.00	10.50	36.80
	(82.40-100.00)	(0.10-26.00)	(33.50-79.80)	(43.50-87.40)	(20.30-66.50)	(24.50-71.10)	(54.40-94.00)	(1.30-33.10)	(16.30-61.60)
Adult	92.30	15.40	23.10	76.90	15.40	76.90	61.50	7.70	69.20
	(63.90-99.80)	(1.90-45.50)	(5.00-53.80)	(46.20-95.00)	(1.90-45.50)	(46.20-95.00)	(31.60-86.10)	(0.20-36.00)	(38.60-90.90)

* Standard deviation values not applicable for lack of data;

¥ Confidence interval at the 95% level.

Helminth abundance and prevalence showed no statistically significant differences between host sexes. However, with regard to host age, *D. longispiculata* abundance and prevalence were significantly higher in young hosts than in adults, while *O. microcephalus* also showed significant differences in abundance and prevalence depending on host age, with higher values in adult hosts than in young hosts (Tables 2 and 3). Abundance analysis for *C. tentaculata* and Cestoda could not be performed because of the low number of occurrences (*C. tentaculata* was found in three females, two adults and one young; and Cestoda was found in only one young female). For *A. raillieti*, *C. tentaculata*, *T. orloffi*, *T. minuta*, *T. turgida*, and *V. hamata*, no statistically significant differences were found regarding the analyzed parameters.

**Table 2:** Mann-Whitney (*U*) and p-value of abundance of the helminths in relation to host sex and age found in *Didelphis marsupialis* (N = 32; N_male_ = 8; N_female_ = 24; N_young_ = 19; N_adult_ = 13) from Mato Grosso State, Brazil.

**Parameters**	***Aspidodera railieti***		***Cruzia tentaculata***		***Didelphonema longispiculata***		***Travassostrongylus orloffi hamata***		***Trichuris minuta***		***Turgida turgida***		***Viannaia***		**Cestoda**		***Oligacanthorhychus microcephalus***	
	*U*	p	*U*	p	*U*	p	*U*	p	*U*	p	*U*	p	*U*	p	*U*	p	*U*	p
Sex	75	0.372	£	£	95	0.981	91	0.843	82.5	0.490	71	0.267	82	0.552	£	£	79	0.227
Age	82.5	0.120	£	£	72.5	0.032*	106	0.509	91.5	0.141	86.5	0.145	82.5	0.116	£	£	90	0.036*

* Significant at the 5% level;

£Not applicable.

**Table 3:** Chi-square and p-value of prevalence of the helminths in relation to host sex and age found in *Didelphis marsupialis* (N = 32; N_male_ = 8; N_female_ = 24; N_young_ = 19; N_adult_ = 13) from Mato Grosso State, Brazil.

**Parameters**	***Aspidodera railieti***		***Cruzia tentaculata***		***Didelphonema longispiculata***		***Travassostrongylus orloffi***		***Trichuris minuta***		***Turgida turgida***		***Viannaia hamata***		**Cestoda**		***Oligacanthorhynchus microcephalus***	
χ^2^	p	χ^2^	p	χ^2^	P	χ^2^	p	χ^2^	p	χ^2^	p	χ^2^	p	χ^2^	p	χ^2^	p
Sex	0.344	0.557	1.103	0.293	0.169	0.681	0.463	0.496	0.194	0.659	2.116	0.146	0.463	0.495	0.344	0.557	0.666	0.414
Age	1.508	0.219	0.930	0.311	3.802	0.032*	0.267	0.599	2.565	0.109	2.795	0.094	1.157	0.282	1.508	0.219	3.239	0.044^*^

* Significant at the 5% level.

With regard to the influence of host body size on helminth abundance or on species richness in each infracommunity, a significant relationship was observed between male host body size and total helminth abundance (beta = 1.5001, t = 3.4217, r^2^ = 0.6612, p = 0.0141). This relationship was not observed in female hosts (beta = 0.5521, t = 0.7639, r^2^ = 0.0258, p = 0.4530). Concerning species richness, no significant relationship was observed either for males (beta = 0.00731, t = 0.8718, r^2^ = 0.1124, p = 0.4168) or females (beta = -0.00603, t = -1.3357, r^2^ = 0.0750, p = 0.1952).

According to the importance indices, the most commonly observed species of the component community, which were present in most infracommunities, were *A. railietti*, *T. orloffi*, and *V. hamata*. The rarest species was *C. tentaculata* (Table 4).

**Table 4:** Importance indices of helminth species found in *Didelphis marsupialis* from Mato Grosso State, Brazil.

**Helminth Specie**	**Importance Indices**	**Classification**
*Aspidodera raillieti*	66.25	Dominant
*Cruzia tentaculata*	<0.01	Subordinate
*Didelphonema longispiculata*	0.49	Co-dominant
*Travassostrongylus orloffi*	4.54	Dominant
*Trichuris minuta*	0.07	Co-dominant
*Turgida turgida*	0.37	Co-dominant
*Viannaia hamata*	15.93	Dominant
Cestoda	0.11	Co-dominant
*Oliganthorhynchus microcephalus*	0.36	Co-dominant

Sampling occurred in two seasonal periods, but the design was for the study by Mendonça et al. (2020) as mentioned, so the sampling was not strong for a seasonal analysis for the occurrence of helminths. The unequal number of animals per season, associated with a 96.9% helminth occurrence in the animals makes any seasonal statistical analysis insignificant, so this analysis will not be detailed.

## Discussion

Herein, we provide the first study of the helminth community structure of the common opossum in the Amazonian Arc in northern Mato Grosso, Brazil. In addition, this study provides the first record of the species *A. raillieti*, *D. longispiculata*, *T. orloffi*, *T. minuta*, *V. hamata*, and *O. microcephalus* in Mato Grosso. Among the helminth taxa (*A. raillieti*, *C. tentaculata*, *T. orloffi*, *T. minuta*, *T. turgida*, *V. hamata*, and *O. microcephalus*), these host–parasite associations were expected.

Previous studies on the helminth fauna of the common opossum include those by Jiménez et al. (2011) in French Guiana, Acosta-Virgen et al. (2015) in Mexico, and Chero et al. (2017) in Peru. Other reports on *D. marsupialis* helminths were species descriptions (Adnet et al., 2009; Chagas-Moutinho et al., 2007). The helminth species richness observed in the present study (nine species) was in accordance with those observed in previous studies that respectively found nine (French Guiana), 11 (Mexico), and 11 (Peru) helminth species in *D. marsupialis* (Jiménez et al., 2011; Acosta-Virgen et al., 2015; Chero et al., 2017). The similarity between observed and expected helminth species richness indicates that all helminth species present in this component community were likely to have been sampled.

In the present study, nematodes constituted 77.8% of all species in the common opossum helminth fauna, showing values similar to those found in other studies in this host (Jiménez et al., 2011; Acosta-Virgen et al., 2015; Chero et al., 2017). The helminth fauna was composed of five helminth species that have direct life cycles (*A. raillieti*, *C. tentaculata*, *T. minuta*, *T. orloffi*, and *V. hamata*), while four have indirect life cycles with transmission by intermediate host ingestion (Cestoda, *D. longispiculata*, *O. microcephalus*, and *T. turgida*). According to Acosta-Virgen et al. (2015), regional differences in the composition and abundance of helminth species could be related to the local availability of parasites (or their intermediate hosts), as well as to the compatibility between the host and the helminth species. Furthermore, *D. marsupialis* is a habitat generalist, with wide variation in adaptation and foraging across environments (Emmons & Feer, 1997; Faria et al., 2019), which may expose this host species to a wide range of parasites along its geographical range.

The most common and abundant species (*A. raillieti*) has previously been observed in studies conducted by Jiménez et al. (2011) and Chero et al. (2017), who also reported *A. raillieti* as having the greatest abundance and prevalence in *D*. *marsupialis* in French Guiana and Peru, respectively. Species of the genus *Aspidodera* are common helminths of the orders Cingulata, Didelphimorphia, Pilosa, and Rodentia (Santos et al., 1990; Jiménez-Ruiz et al., 2006), and are commonly reported to infect opossums of the genus *Didelphis* (Silva & Costa, 1999; Antunes, 2005; Chero et al., 2017; Costa-Neto et al., 2019).

*Viannaia hamata* was the second most abundant and prevalent species, corroborating studies on helminths in *D. albiventris* (Silva & Costa, 1999) and *D. aurita* (Costa-Neto et al., 2019) in the states of Minas Gerais and Rio de Janeiro, respectively. However, and rather surprisingly, *C. tentaculata*, which was found in low abundance and as having low prevalence in the present study, was commonly reported as one of the most abundant species infecting *D. marsupialis* (Jiménez et al., 2011; Chero et al., 2017), *D. albiventris* (Silva & Costa, 1999; Cirino et al., 2022), and *D. aurita* (Costa-Neto et al., 2019).

*Travassostrongylus orloffi* was the third most abundant species, although it was not found in either Jiménez et al. (2011) or in Chero et al. (2017). Acosta-Virgen et al. (2015) suggested that the species of the genus *Travassolstrongylus* found in *D. marsupialis* in Mexico could be *T. orloffi.* This species was also recorded in *D. aurita* (Costa-Neto et al., 2019) and *D. albiventris* (Silva & Costa, 1999; Gomes et al., 2003; Antunes, 2005).

In general, studies of mammalian parasites have shown higher rates of infection in male than in female hosts (Zuk & McKean, 1996; Poulin, 2007). Although no significant differences were observed in the abundance and prevalence of helminths between male and female hosts, for most species, the abundance indices were higher in females. However, the absence of a significant difference may be due to the large variation in abundance among hosts, which can be seen in the high values of the standard deviation. Castro et al. (2017) and Costa-Neto et al. (2019), who studied another species of the genus *Didelphis*, *D. aurita*, found significant differences in parasitological indices between male and female hosts only for *O. microcephalus*, whose prevalence was higher in females, and for *T. orloffi*, whose prevalence and abundance were higher in males. Cirino et al. (2022), studying the helminth fauna of *D. albiventris*, found a significant difference in host sex only for the nematode *C. tentaculata*, which was more abundant in females. This species was found only in female hosts in the present study, but in low abundance and prevalence.

With regard to host age, *O. microcephalus* showed a higher abundance and prevalence in adult hosts, consistent with the notion of a gradual increase in infection throughout the host lifespan. In contrast, the greater abundance and prevalence of *D. logispiculata* in young hosts can be attributed to early infections of this helminth, which has previously been observed in helminths in other mammals (Cardoso et al., 2019; Lucio et al., 2021). Larger hosts are expected to have a larger number of parasites and more parasitic species (Guégan et al., 1992; Morand & Poulin, 1998). Host body size is considered one of the most important determinants of parasite species richness (Kamiya et al., 2014), as larger hosts provide a larger space for parasites than smaller hosts. In addition, large hosts have a larger intake of food than small hosts, which may increase their chances of acquiring parasites. Our results suggest an increase in parasite abundance with increasing host body size in males. However, no relationship was found between host body size and parasite abundance or species richness in female hosts. A similar relationship was observed in another neotropical marsupial, *Metachirus myosuros* (Cirino et al., 2020), in which the authors observed an increase in parasite abundance and species richness with increasing body size.

The community structure of the helminths in the common opossum indicated that there were three core species that were present in most infracommunities: *A. railietti, T. orloffi*, and *V. hamata*. Compared with the helminths of *D. marsupialis* by Jiménez et al. (2011), only *A. raillieti* could be considered a dominant species. *C. tentaculata*, despite being a subordinate species in the present study and reported to have low prevalence and abundance by Jiménez et al. (2011), was reported to be a central species in helminth community studies of *D. aurita* (Costa-Neto et al., 2019) and *D. albiventris* (Cirino et al., 2022). Nevertheless, *A. raillieti* and *V. hamata* were considered the core species in these two studies. *T. orloffi* was reported as a core species by Costa-Neto et al. (2019); however, they suggested that they may act as a local opportunistic species.

The results indicated that the helminth fauna of *D. marsupialis* in southern Amazonia, a transition area with the Cerrado biome, was similar to the helminth fauna observed in other regions and to the congener species *D. aurita* and *D. albiventris*. Host sex was not related to abundance or prevalence. However, host age influenced the abundance and prevalence of acanthocephalans and the nematode *D. longispiculata*. Host body size was a predictor of total helminth abundance in male hosts. This is a novel study of the helminth fauna and helminth community structure in the common opossum in Brazil.

## Appendix 1 Voucher specimens deposited in the Helminthological Collection of the Oswaldo Cruz Institute (CHIOC) and in the Zoological Collection of the Federal University of Mato Grosso (UFMT).

**Table:**

**Species**	**Collection number**
*Aspidodera raillieti*	CHIOC 39310
*Cruzia tentaculata*	CHIOC 39311
*Didelphonema longispiculata*	CHIOC 39190
*Travassostrongylus orloffi*	CHIOC 39312
*Trichuris minuta*	CHIOC 39313
*Turgida turgida*	CHIOC 39314
*Viannaia hamata*	CHIOC 39315
*Oliganthorhynchus microcephalus*	CHIOC 39309
*Didelphis marsupialis*	UFMT 4276, 4277, 4278, 4279, 4280, 4281, 4282, 4283, 4284, 4285, 4286, 4287, 4288, 4289, 4290, 4327, 4328, 4329, 4330, 4331, 4332, 4652, 4653, 4654, 4655, 4697, 4698, 4699, 4700, 4701, 4702, 4703.
